# Evaluation of Biomedical Applications for Linseed Extract: Antimicrobial, Antioxidant, Anti-Diabetic, and Anti-Inflammatory Activities In Vitro

**DOI:** 10.3390/jfb14060300

**Published:** 2023-05-28

**Authors:** Mohamed M. Alawlaqi, Aisha M. H. Al-Rajhi, Tarek M. Abdelghany, Magdah Ganash, Hanan Moawad

**Affiliations:** 1 Biology Department, College of Science, Jazan University, Jazan 82817, Saudi Arabia; 2 Department of Biology, College of Science, Princess Nourah bint Abdulrahman University, P.O. Box 84428, Riyadh 11671, Saudi Arabia; 3 Botany and Microbiology Department, Faculty of Science, Al-Azhar University, Cairo 11725, Egypt; tabdelghany.201@azhar.edu.eg; 4 Biology Department, Faculty of Science, King Abdulaziz University, Jeddah 21589, Saudi Arabia; mganash@kau.edu.sa; 5 Plant Department, Faculty of Science, Fayoum University, Fayoum 63514, Egypt; hananmoawad200@gmail.com

**Keywords:** linseed, phenolic, flavonoid, methicillin-resistant *Staphylococcus aureus*, antioxidant, anti-diabetic, anti-inflammatory

## Abstract

Background: In the last few decades, the development of multidrug-resistant (MDR) microbes has accelerated alarmingly and resulted in significant health issues. Morbidity and mortality have increased along with the prevalence of infections caused by MDR bacteria, making the need to solve these problems an urgent and unmet challenge. Therefore, the current investigation aimed to evaluate the activity of linseed extract against Methicillin-resistant *Staphylococcus aureus* (MRSA) as an isolate from diabetic foot infection. In addition, antioxidant and anti-inflammatory biological activities of linseed extract were evaluated. Result: HPLC analysis indicated the presence of 1932.20 µg/mL, 284.31 µg/mL, 155.10 µg/mL, and 120.86 µg/mL of chlorogenic acid, methyl gallate, gallic acid, and ellagic acid, respectively, in the linseed extract. Rutin, caffeic acid, coumaric acid, and vanillin were also detected in the extract of linseed. Linseed extract inhibited MRSA (35.67 mm inhibition zone) compared to the inhibition zone (29.33 mm) caused by ciprofloxacin. Standards of chlorogenic acid, ellagic acid, methyl gallate, rutin, gallic acid, caffeic acid, catechin, and coumaric acid compounds reflected different inhibition zones against MRSA when tested individually, but less than the inhibitory action of crude extract. A lower MIC value, of 15.41 µg/mL, was observed using linseed extract than the MIC 31.17 µg/mL of the ciprofloxacin. The MBC/MIC index indicated the bactericidal properties of linseed extract. The inhibition % of MRSA biofilm was 83.98, 90.80, and 95.58%, using 25%, 50%, and 75%, respectively, of the MBC of linseed extract. A promising antioxidant activity of linseed extract was recorded, with an IC_50_ value of 20.8 µg/mL. Anti-diabetic activity of linseed extract, expressed by glucosidase inhibition, showed an IC_50_ of 177.75 µg/mL. Anti-hemolysis activity of linseed extract was documented at 90.1, 91.5, and 93.7% at 600, 800, and 1000 µg/mL, respectively. Anti-hemolysis activity of the chemical drug indomethacin, on the other hand, was measured at 94.6, 96.2, and 98.6% at 600, 800, and 1000 µg/mL, respectively. The interaction of the main detected compound in linseed extract (chlorogenic acid) with the crystal structure of the 4G6D protein of *S. aureus* was investigated via the molecular docking (MD) mode to determine the greatest binding approach that interacted most energetically with the binding locations. MD showed that chlorogenic acid was an appropriate inhibitor for *S. aureus* via inhibition of its 4HI0 protein. The MD interaction resulted in a low energy score (−6.26841 Kcal/mol) with specified residues (PRO 38, LEU 3, LYS 195, and LYS 2), indicating its essential role in the repression of *S. aureus* growth. Conclusion: Altogether, these findings clearly revealed the great potential of the in vitro biological activity of linseed extract as a safe source for combatting multidrug-resistant *S. aureus*. In addition, linseed extract provides health-promoting antioxidant, anti-diabetic, and anti-inflammatory phytoconstituents. Clinical reports are required to authenticate the role of linseed extract in the treatment of a variety of ailments and prevent the development of complications associated with diabetes mellitus, particularly type 2.

## 1. Introduction

In the clinical management of infections, multidrug resistance (MDR) among pathogenic bacteria is on the rise globally [1]. A significant rise in pathogenic bacterial resistance levels and a sharp decline in the effectiveness of traditional antibacterial therapy were frequently witnessed across the entire spectrum of available therapeutics [2]. Antibiotic resistance is present in more than 90% of staphylococci, pneumococci, and enterococci isolated from severe infections. The prevalence of multidrug-resistant bacteria, including methicillin-resistant *Staphylococcus aureus* (MRSA), β-lactam- and macrolide-resistant pneumococci, and glycopeptide- and vancomycin-resistant enterococci, has been previously recorded [3]. 

Natural products from plants can be used as an antimicrobial agent, constituting an alternative approaches available to decrease the dose-dependent side effects of commercially available traditional antibiotics and minimize antibiotic resistance [4,5,6,7,8].

Further, several biological activities were also associated with the natural products of plant origin. These activities include inflammasome minimization caused by microbial infection [9]—which represses the development of cancer cell proliferation [10]—and may apply to fabricated material used as wound dressings [11].

Flax (*Linum usitatissimum* L.), an annual plant belonging to the Linaceae family that thrives in temperate and Mediterranean climate zones, may be a rich source of biologically active constituents [4]. *L. usitatissimum,* also known as linseed, is an economically vital crop globally. Several benefits, both therapeutic and dietary, have been associated with flax seeds, as represented by seedcake. Seedcake has been used to eliminate many illnesses including respiratory tract, skin, and gastrointestinal tract infections [12]. Antioxidant biological activities have been reported in seedcake extract, [13,14], due to the presence of several phenolic contents such as *p*-coumaric acid, caffeic acid, and ferulic acid, and their glycosyl derivatives [15]. As mentioned in a previous study, carotenoids, flavonoids, tannins, vitamins E, phenols, flavonoids, and phenolic compounds, as contents of linseed, play an important role in several biological activities, for instance, antioxidant, anti-diabetic, and anti-inflammatory activities [16]. Management of gastrointestinal and diarrheal infections was documented by *L. usitatissimum* via inhibition of enteric and non-enteric pathogens [17]. Akl et al. [14] evaluated the toxicity and antimicrobial activity of flaxseed extract and indicated that it is effective against only four of seven strains of bacteria but has no toxicity towards human tumor cell lines; lung and colon carcinoma cell lines were inhibited by extract with IC_50_ values of 22.6 and 22.3 μ/mL, respectively; moreover, flaxseed extract has no anticoagulating potential. In recent reports, aqueous flaxseed extract and its oil extract reflected antibacterial effectiveness against *Streptococcus mutans* [18].

The medical approach still faces difficulties in managing diabetes with drugs that have no side effects. Due to this worry, there is now more demand for safe, all-natural diabetes treatments derived from herbs. As mentioned in the literature review by Ma et al. [19], with over 693 million cases anticipated by 2045, diabetes mellitus poses a severe threat to world health. Anti-inflammatory and anti-diabetic effects of an aqueous extract of flaxseed were reported in vitro and in vivo in an investigational model of rats [20]. Remarkable antibacterial activity of *L. usitatissimum* was recorded by Haroon et al. [21] against *S. aureus* and other bacterial species isolated from diabetic patients; owing to this activity, Haroon et al. [21] suggested the application of linseed-coated bandages for diabetic foot infection treatment. Therapeutic action of linseeds, including analgesic, antioxidant, and anti-inflammatory action, has been reported to lower the glucose level in the blood and reduce the human body’s resistance to insulin [22].

Mohamed et al. [23] reported that bakery products supplemented with flaxseed possess efficacy for reducing blood glucose and serum lipids in type 2 diabetic patients. Sanaa et al. [24] documented the anti-hypertrophic potential of flaxseed lignans to prevent hypertrophy of cardiac muscle by controlling myocardial remodeling and oxidative stress.

The study of interactions and binding affinities for biological activity of proteins and peptides is frequently carried out using in silico tools such as molecular docking (MD). Various proteins, such as receptors, enzymes, hormones, and antibodies, rarely function in the cell by themselves; instead, they interact with a variety of partners, including small molecules [25]. MD was designed to simulate these interactions at the molecular level by forecasting the 3D structures of complexes. Through MD, investigators can predict the binding site and pose of a protein with its partner, revealing the relationship between protein structure and function and assisting drug design in a variety of ways [26].

Generally, biological activities may differ, even for the same type of plant, based on cultivation and soil conditions; therefore, we also carried out chemical characterization of linseed extract. Many diabetics suffer from microbial infections in the foot, and resistance of these microbes to antibiotics poses a problem; as a result, our study focused on an antibiotic-resistant strain isolated from diabetic foot infection patients. Furthermore, the goals of our studies included evaluating antioxidant and anti-inflammatory activities.

## 2. Materials and Methods

### 2.1. Source of Chemicals

Analytical grade chemicals were obtained from Sigma-Aldrich, Taufkirchen, Germany. These included DPPH (2,2-diphenyl-1-picrylhydrazyl), Dimethyl sulfoxide (DMSO), ascorbic acid, trypan blue dye, crystal violet, L-glutamine, 25% Trypsin-EDTA, gentamycin, and bacterial growth medium. Active Fine Chemicals Limited, Dhaka, Bangladesh, was the source of solvents and reagents.

### 2.2. Plant Sample and Test Microbe

The linseeds were obtained from Jazal Arabia Trading Company, Riyadh, Saudi Arabia. Methicillin-resistant *Staphylococcus aureus* (MRSA) was obtained from Ain Shams University Hospital, Cairo, Egypt. Test subjects were selected from the most severe cases of diabetic foot infection with a higher rate of therapeutic failure. The samples of diabetic foot infection were collected via an ethics committee, and the authors only obtained the bacterial isolate after confirmation of methicillin resistance compared to other sensitive strains. According to the hospital laboratory, eight samples were collected and five samples showed the presence of MRSA.

### 2.3. Preparation of Linseed Extraction

The linseeds were milled using a mechanical grinder; then, the ground seeds were macerated for 24 h in methanol under shaking conditions, filtrated, and concentrated at 40 °C via a rotary evaporator (IKA, Berlin, Germany). The obtained extract was kept at 4 °C for further analysis and evaluation of biological activities.

### 2.4. Detection of Phenolic and Flavonoid Content of Linseed Extract by HPLC

An amount of 5 μL of the linseed extract was injected into the HPLC apparatus (Agilent 1260 series, Agilent Technologies, Santa Clara, CA, USA). HPLC was characterized with the Eclipse C18 column (4.6 mm × 250 mm i.d., 5 μm), maintained at 40 °C. A total of 2 buffers were used as a mobile phase at constant flow rate (0.9 mL/minute). The first buffer was 0.05% trifluoroacetic acid with Milli-Q water while the second buffer was 0.05% trifluoroacetic acid with acetonitrile. Following a 20 min run for the mobile phase, the serial dilution results were 82% (0 min), 80% (0–5 min), 60 % (5–8 min), 60% (8–12 min), 82% (12–15 min), 82% (15–16 min), and 82% (16–20 min) of the first buffer. The phenolic and flavonoid constituents were recorded at a wavelength 280 nm via an ultraviolet (UV) detector (Icon Scientific Inc., North Potomac, MD, USA). The quantity of each constituent was recognized based on the introduced standard compounds [27].

### 2.5. Antibacterial Activity of Linseed Extract and Standard of Penolic and Flavonoid Compounds against Methicillin-Resistant S. aureus

The activity of linseed extract against the tested MRSA was performed according to the recommendations of the Clinical and Laboratory Standards Institute (CLSI) with some modifications. The antibacterial activity was performed via the agar diffusion technique using Mueller–Hinton agar (MHA) plates. MHA was inoculated via seedling of MRSA in the amount of 2 × 10^8^ cfu/mL, and a 6 mm-diameter depression was made in the inoculated MHA then filled with the extract (100 µL of 100 µg/mL). The cultivated plates were incubated at 37 °C for 24 h. At the end of the incubation time, the appearance of an inhibition zone was recorded. The extracted solvent was used as a negative control, while the antibiotic (100 µL of 5 µg Ciprofloxacin) was applied as a positive control [7]. Standards of chlorogenic acid, ellagic acid, methyl gallate, rutin, gallic acid, caffeic acid, catechin, coumaric acid, and vanillin (100 µL of 100 µg/mL) were tested against MRSA, as mentioned in the case of linseed extract.

### 2.6. Detection of Minimal Inhibitory Concentration (MIC) of Linseed Extract

Linseed extract was tested to determine its MIC against MRSA using the Mueller–Hinton broth method of microdilution broth. Various serial dilutions were created with 0.98 to 1000 µg/mL of linseed extract. In sterile 96-well polystyrene microtitrate plates, 200 μL per well of each appropriate dilution of linseed extract was dispensed. Fresh MRSA culture inoculum was made in 0.85% sterile NaCl to match the required 1.0 McFarland turbidity. After adding 2 µL of MRSA inoculum, each well received a final dose of 3 × 10^6^ colony-forming units/mL. After the incubation period, the MIC was assessed by the unaided eye, reflecting full inhibition (clear medium broth) of MRSA growth. The inoculum of MRSA without linseed extract was utilized as a positive control, while linseed extract without inoculum of MRSA was applied as a negative control in every microplate [28].

### 2.7. Detection of Minimum Bactericidal Concentration (MBC) of Linseed Extract

MBC was performed by plating 100 mL of the MRSA colonies from each well, showing complete inhibition, and from the last positive and growth control, showing complete inhibition from the previous positive and growth control onto the MHA plates. After the incubation period, the MBC was visually assessed, confirming the reproduction of the inhibition of MRSA growth by the lowest concentration of linseed extract. To demonstrate the bactericidal or bacteriostatic effect of linseed extract, the MBC/MIC ratio was calculated. If MBC/MIC was not greater than four times the MIC, the extract possessed bactericidal efficacy [29].

### 2.8. Assessment of Linseed Extract against Antibiofilm of Methicillin-Resistant S. aureus

Antibiofilm activity was detected via microtiter plate test in 96-well polystyrene flat-bottom plates containing fresh trypticase soy yeast broth (TSY) (300 μL in each well), and amended with the sublethal doses (75, 50, and 25% of MBC of linseed extract). The plates were inoculated with 10^6^ CFU/mL of MRSA. At 37 ºC, the plates were incubated for 48 h, and then the supernatant broth was detached, followed by washing with sterile distilled water (SDW) to eliminate free-floating bacterial cells. Then, the plates were air-dried for 30 min, and the formed MRSA biofilm was stained utilizing 0.1% solution of crystal violet (CV) dissolved in SDW for 17 min at 25 °C. The excess CV was detached; then, the plates were washed three times with SDW. A volume (250 μL) of ethanol (95%) was added in each well to solubilize the linked dye to MRSA cells. After 15 min of the incubation period, the absorbance (Ab) was recorded at a wavelength of 570 nm using a microplate reader. The biofilm inhibition (BI) of MRSA was calculated based on the following formula:

$$\%\mathrm{BI}\;=1-\left(\frac{\mathrm{Ab}.\;\mathrm{treated}\;–\;\mathrm{Ab}.\;\mathrm{Blank}}{\mathrm{Ab}.\;\mathrm{control}\;–\;\mathrm{Ab}.\;\mathrm{Blank}}\right)\times 100$$

Blank represented the absorbance of media only; treatment with linseed extract represented the absorbance of MRSA from treatment. In contrast, control represented the absorbance of MRSA without linseed extract treatment [30,31].

### 2.9. Antioxidant Potential of Linseed Extract via DPPH Method

The ability of linseed extract to neutralize free radicals was examined using DPPH. Linseed extract in ethanol was prepared with a 0.1 mM DPPH solution, and 1 mL of this solution was then added to 3 mL of the extract at various concentrations (3.9–1000 µg/mL). In this case, only ethanol extracts were used, and different dilutions of those extracts were prepared using the dilution method. After shaking the mixture well, it was allowed to sit for 30 min at room temperature. Then, at 517 nm, the absorbance was measured with a spectrophotometer (UV-Vis Milton Roy). Three different iterations of the experiment were carried out with ascorbic acid as the standard reference substance. The sample’s IC_50_ value, the quantity of linseed extract required to inhibit 50% of the DPPH free radical, was calculated using a log dose-inhibition curve [32]. To calculate the percent DPPH scavenging result, the following equation was used:

$$\mathrm{DPPH}\;\mathrm{scavenging}\;\left(\%\right)=\frac{\mathrm{Ab}.\;\mathrm{control}\;–\;\mathrm{Ab}.\;\mathrm{in}\;\mathrm{presence}\;\mathrm{of}\;\mathrm{tested}\;\mathrm{sample}\;}{\mathrm{Ab}.\;\mathrm{control}\;}\times 100$$

### 2.10. Preparation of Erythrocyte Suspension and Hypotonicity Induced Haemolysis

An amount of 3 mL of fresh blood samples from a healthy human (the author) were collected in heparinized tubes, followed by centrifugation at 3000 rpm for 10 min. An amount of normal saline was added to the supernatant (*v*/*v*) to dissolve the blood pellets. The obtained volume of the dissolved blood pellets was reconstituted as a 40% *v*/*v* suspension using a solution of isotonic buffer, named sodium phosphate (10 mM), with pH 7.4. The solution of buffer composed of NaH_2_PO_4_ (0.2 g), Na_2_HPO_4_ (1.15 g), and NaCl (9 g) was placed in a liter of distilled water (DW). The resuspended supernatant was utilized as such. Linseed extract was dissolved in DW as a hypotonic solution. An amount of 5 mL of the hypotonic solution containing different concentrations of the linseed extract, ranging 100–1000 μg/mL, was placed in centrifuge tubes. An isotonic solution (5 mL) containing different concentrations of the linseed extract, ranging from 100–1000 μg/mL, was also placed in centrifuge tubes. Tubes containing 5 mL of the DW and 5 mL of the prepared indomethacin (200 μg/mL) were used as a control. The supernatant of 0.1 mL of the prepared erythrocyte suspension was added to all tubes separately and mixed gently. At 37 °C for 1 h, the mixtures were incubated and, subsequently, centrifuged at 1300 g for 3 min. Via a Spectronic (Milton Roy) spectrophotometer at 540 nm, the Ab of the supernatant containing the hemoglobin was assessed. The % of hemolysis was designed by assuming that he hemolysis generated in the presence of DW was 100%. The hemolysis inhibition (HI) was calculated based on the following formula:

$$\%\;\mathrm{HI}\;=1-\left(\frac{\mathrm{Ab}.\;2-\;\mathrm{Ab}.\;1}{\mathrm{Ab}.\;3\;–\;\mathrm{Ab}.\;1}\right)\times 100$$

Ab.1 indicates the Ab of linseed extract in an isotonic solution, Ab.2 indicates the Ab of linseed extract in a hypotonic solution, while Ab.3 indicates the Ab of the control sample in hypotonic solution [33].

### 2.11. Anti-Diabetic Activity of Linseed Extract via α-Glucosidase Inhibitory Assessment

Linseed extract was assessed for inhibitory activity of α-glucosidase using the procedure of Pistia Brueggeman and Hollingsworth [34] with minor modifications. Different concentrations of linseed extract, ranging from 1.97 to 1000 μg/mL, were mixed with 10 μL of the α-glucosidase solution (1 U/mL), then incubated at 37 °C for 20 min with a supplementary amount of 125 μL phosphate buffer (0.1 M, pH 6.8). Following 20 min of incubation, the reaction mixture was initiated by adding 1 M of 4-nitrophenyl β-d-glucopyranoside (20 μL) as substrate, and the reaction mixture was kept for 30 min. For terminating the reaction, 50 μL of 0.1 N Na_2_CO_3_ was added, and the final Ab was measured at 405 nm utilizing a Biosystem 310 joined spectrophotometer. α-Glucosidase inhibition (GI) was calculated based on the following formula:

$$\%\;\mathrm{GI}\;=\frac{\mathrm{Ab}\;\mathrm{Blank}\;-\;\mathrm{Ab}\;\mathrm{linseed}\;\mathrm{extract}\;\mathrm{treatment}\;}{\mathrm{Ab}\;\mathrm{Blank}\;}\times 100$$

The amount of enzyme (α-glucosidase) needed to produce one mol of the product (*p*-nitrophenol) from the substrate (4-nitrophenyl β-d-glucopyranoside) per minute can be considered one unit of the enzyme. The regression equation used to calculate the IC_50_ (concentration of linseed extract needed to inhibit enzyme activity at a 50% level) was obtained by plotting linseed extract concentration (1.97–1000 µg/mL) and % inhibition for linseed extract.

### 2.12. Molecular Docking Methodology

The ligand was permitted to be flexible to determine the proper conformations and produce structures with the least possible amount of energy. After docking, the optimal conformations of the ligand were examined for their binding interactions.

In this study, an effort was made to use the MOE (Molecular Operating Environment) software tool to carry out the docking of chlorogenic acid into 4G6D protein. To envision the interactions between *S. aureus* 4G6D and Ligand, LigPlot developed in MOE was used.

Preparation of ligand: the structures of chlorogenic acid were constructed using PerkinElmer ChemOffice Suite 2015. Furthermore, related 3D structures emerged, and the detected molecules’ energies were reduced using the default MOE energy minimization algorithm parameters (gradient: 0.05, force field: MMFF94X).

Preparation of receptor protein: *S. aureus* 4G6D, the protein molecule utilized throughout our investigation, was obtained from Protein Data Bank (http://www.rcsb.org/pdb accessed on 19 July 2022). After the water molecules were removed, the protein molecule underwent 3D protonation. Using the default settings for the MOE energy minimization algorithm (gradient: 0.05, force field: MMFF94X), the energy of the restored protein molecule was reduced to the absolute minimum. To evaluate the MOE-Dock program’s accuracy, the cocrystallized ligand was removed from the active site and redocked within the inhibitor binding cavity of *S. aureus* 4G6D.

### 2.13. Statistical Study

The obtained records were investigated via SPSS version 15.0 (SPSS Inc., Chicago, IL, USA). The whole values are expressed as ± standard deviation (SD) of the mean values [9].

## 3. Results and Discussion

### 3.1. Phenolic and Flavonoid Characterization of Linseed

**Figure 1:**
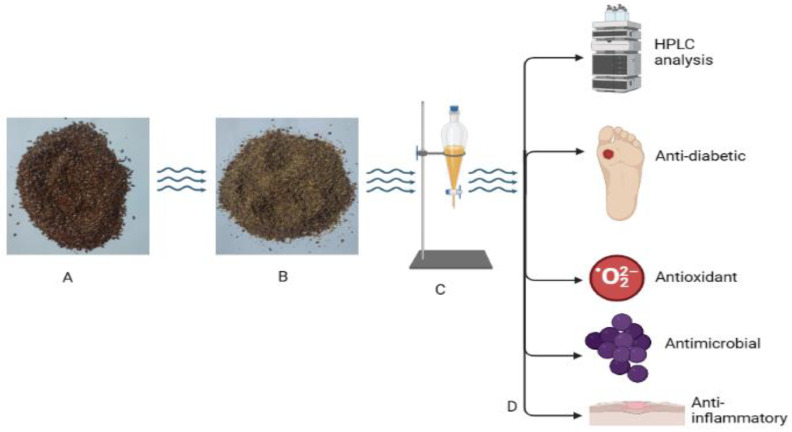
Ground linseed and various relevant evaluations to identify the phytoconstituents and other biological activities of the extracted product. Linseed (**A**), grinded linseed (**B**), Extraction process (**C**,**D**) further analysis biological processes of linseed extract. This figure was created with BioRender.com, 14 April 2023.

**Figure 2:**
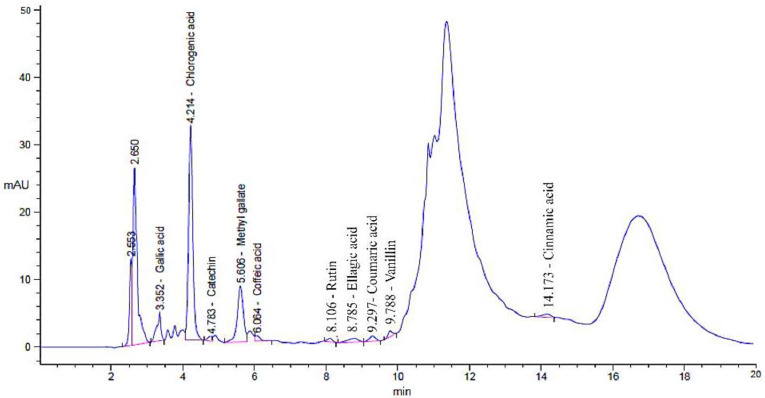
High-performance liquid chromatography chromatograms of detected flavonoid and phenolic compounds in linseed extract.

**Figure 3:**
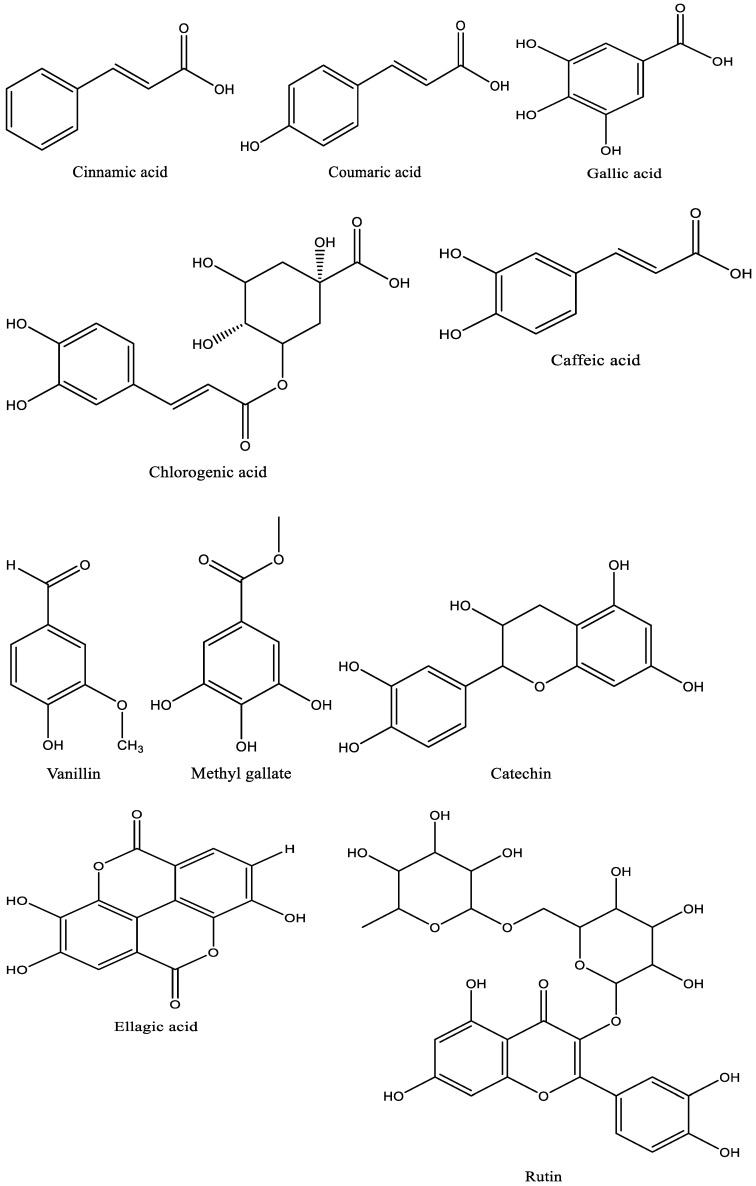
Chemical construction of identified phenolic and flavonoid compounds in linseed extract.

The ground linseed was subjected to phenolic and flavonoid detection and several biological applications (Figure 1). Analysis of linseed via HPLC reflected the presence of phenolic as well as flavonoid compounds. These compounds were identified with different areas (%), different retention times (Table 1 and Figure 2), and different chemical instructions (Figure 3). A high content of chlorogenic acid (1932.20 µg/mL) was recorded in linseed extract. In comparison, a moderate content of methyl gallate, gallic acid, and ellagic acid was recorded (284.31 µg/mL, 155.10 µg/mL, and 120.86 µg/mL, respectively) in the linseed extract. Rutin, coffeic acid, coumaric acid, and vanillin were detected in low concentrations of 32.81 µg/mL, 32.78 µg/mL, 17.02 µg/mL, and 16.45 µg/mL, respectively. A very low concentration, 8.84 µg/mL, was recorded for cinnamic acid. Unfortunately, several biologically active compounds, including ferulic acid, naringenin, daidzein, quercetin, apigenin, kaempferol, and hesperetin, were not detected in the linseed extract. Linseed’s phenolic and flavonoid compounds may differ according to cultivar, varietal genetic makeup, and climatic condition in the cultivation regions. Therefore, Yaqoob et al. [35] documented the presence of gallic acid, hydroxybenzoic acid, coumaric acid, ferulic acid, caffeic acid, cinnamic acid, and benzoic acid in several cultivars of linseed via HPLC, but at different levels according to cultivar. In the current decade, there has been increasing attention paid to flavonoids and phenolic molecules due, in particular, to their antioxidant ability and likely benefits in pharmaceutical and food applications [8,36,37].

### 3.2. Antimicrobial Activity of Linseed Extract and Standard of Penolic and Flavonoid Compounds against MRSA

**Figure 4:**
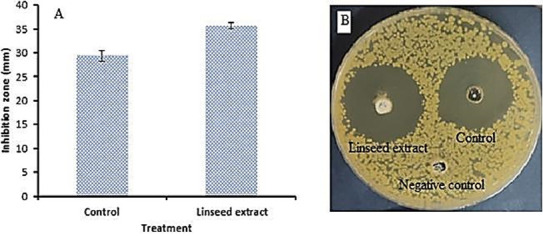
Antibacterial activity of linseed extract against Methicillin-resistant *S. aureus,* indicated by chromatogram (**A**) and well-diffusion method (**B**).

**Figure 5:**
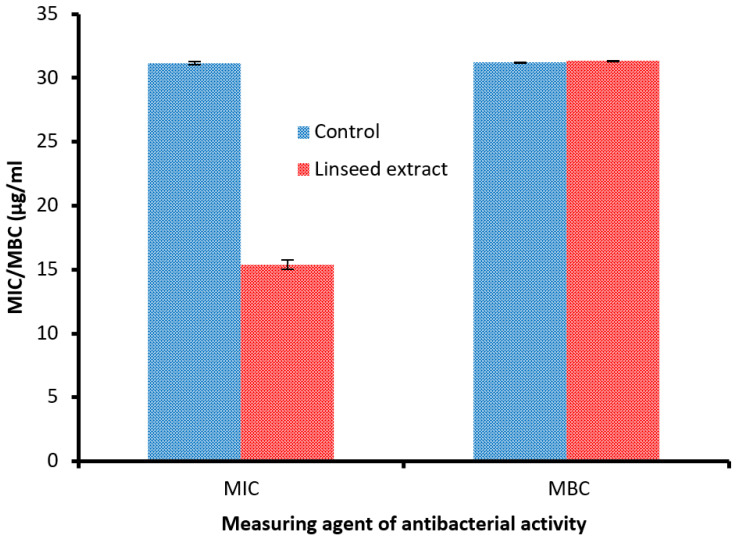
MIC and MBC of linseed extract against Methicillin-resistant *S. aureus*.

Linseed extract was tested against Methicillin-resistant *S. aureu* (MRSA) in diabetic foot infection patient colonization (Figure 4). Linseed extract showed inhibitory activity, with a 35.67 ± 1.15 mm inhibition zone compared to the control’s 29.33 ± 0.58 mm inhibition zone. Furthermore, the MIC (15.41 ± 0.36 µg/mL) of the linseed extract was less than the MIC (31.17 ± 0.14 µg/mL) of the control, indicating its potent activity towards MRSA (Figure 5).

**Figure 6:**
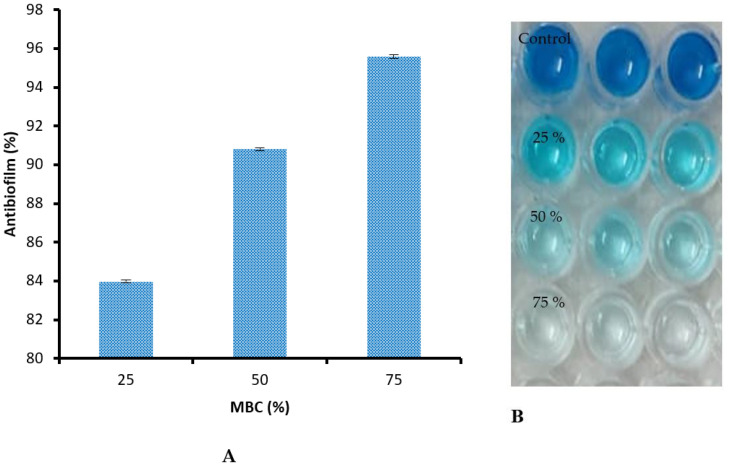
Antibiofilm activities of linseed extract against Methicillin-resistant *S. aureus* (**A**) and stained biofilm color in the microtiter plate depended on biofilm viability (**B**).

Via MBC detection, there were very negligible differences between linseed extract (31.32 ± 0.03 µg/mL) and control (31.22 ± 0.03 µg/mL) (Figure 5), but the MBC/MIC index of linseed extract and control were 2 and 1, respectively, indicating the bactericidal properties of linseed extract, due to its MBC/MIC indexing not being above 4 times its MIC. Haroon et al. [21] showed that *E. coli*, *S. aureus,* and *K. pneumonia* were susceptible to linseed extract with MIC values ranging from 10 to 120 mg/mL. The bactericidal potential of linseed extract may be due to its hydrophobicity; these characteristics may deactivate transport proteins and microbial adhesion, resulting in a disturbance of the cell membrane of bacteria [38,39]. Alternatively, the biological activity of linseed extracts could be attributed to the presence of active constituents that inhibit MRSA. Our results were in agreement with Sultana et al. [40], who documented the antibacterial potential of linseed extract against *S. aureus, Bacillus cereus, Klebsiella pneumoniae,* and *Pseudomonas aeruginosa.* The antibiofilm activity of linseed extract against MRSA was examined using different levels of MBC in linseed extract. As mentioned previously, microbial biofilms cause chronic diseases because they display amplified resistance to antimicrobial compounds and fight phagocytosis. Therefore, the inhibition of bacterial biofilm is considered a main drug target for the management of numerous infections caused by bacteria. Figure 6A shows good values of antibiofilm activity, at 83.98%, 90.80%, and 95.58% at 25%, 50%, and 75% of MBC of linseed extract, respectively. Furthermore, our observation of stained biofilm color in the microtiter plate depended on biofilm viability (Figure 6B). Antibiofilm activity of linseed extract was recorded against several bacteria that found on the bandages of diabetic foot infection ulcers [21].

In the present investigation, standards of the detected phenolic and flavonoid compounds were tested separately against MRSA, producing different sizes of inhibition zones. Chlorogenic acid produced the largest inhibition zone (17.67 ± 0.58 mm), followed by ellagic acid (17.50 ± 0.87 mm), methyl gallate (15.33 ± 1.15 mm), rutin (15.17 ± 0.76 mm), gallic acid (15.17 ± 0.29 mm), caffeic acid (14.83 ± 1.04 mm), catechin (12.84 ± 1.04 mm), and coumaric acid (10.67 ± 1.15 mm), while vanillin did not demonstrate inhibitory action against MRSA (Table 2). All anti-MRSA activity of these phenolic and flavonoid compounds was compared to that of the antibiotic, which demonstrated a 29.33 ± 0.58 mm inhibition zone. From the obtained finding, the crude extract demonstrated larger inhibition zones compared to any phenolic or flavonoid compounds tested separately; this may be attributed to the extract containing a mixture of these compounds, or because it contains other biologically active compounds. In earlier reports, several phenolic and flavonoid compounds were tested against MRSA; some of these reflected inhibitory potential, including ellagic acid [41,42], ferulic acid, *p*-coumaric acid, caffeic acid [43], gallic acid, chlorogenic acid, *p*-coumaric acid, and caffeic acid [44].

### 3.3. Antioxidant Activity of Linseed Extract

As shown in Table 3, strong antioxidant activity was associated with linseed extract. The antioxidant potential increased with the concentration increment of linseed extract in a concentration-dependent manner; this association began at 1.95 µg/mL, with DPPH scavenging of 27.5%. DPPH scavenging increased to 74.6% at 250 µg/mL and 89.9% at 1000 µg/mL. At high concentrations, e.g., of 500 and 1000 µg/mL of linseed extract, there was a slight decrease in DPPH scavenging % in linseed extract compared to the standard drug ascorbic acid. The IC_50_ of ascorbic acid was low (4.81 g/mL), but a promising IC_50_ value of 20.8 µg/mL was recorded using linseed extract. The antioxidant activity of linseed extract was documented using several solvents, resulting in different levels of activity, ranging from 35.68–66.76% radical scavenging activity (RSA) towards DPPH; acetone extract was the most effective, followed by ethyl alcohol, while water extract was the least effective in the RSA [45]. Akl et al. [14] reported that phenolic compounds of linseed extract reflected great antioxidant potential. Further, from the investigation of phenolic, tannin, flavone, and flavonoid compounds in linseed, Nakhlawy [16] and Yaqoob et al. [35] determined that these extracts had antioxidant properties for therapeutic health functions and food applications.

### 3.4. Anti-Diabetic Activity of Linseed Extract

**Figure 7:**
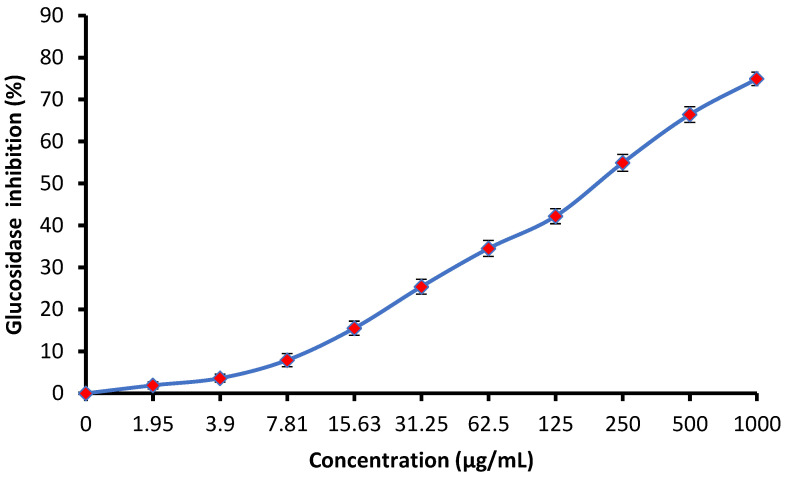
Anti-diabetic activity via measurement of glucosidase inhibition of linseed extract.

The anti-diabetic activity of linseed extract was estimated by measuring glucosidase inhibition (Figure 7). It is clear that glucosidase inhibition increased as linseed extract concentration increased. At 250, 500, and 1000 µg/mL of the extract, the glucosidase inhibition was 54.9, 66.4, and 74.9%, respectively, with an IC_50_ of 177.75 µg/mL, indicating its potential to manage diabetic illnesses. Our result was in line with the findings of Mechchate et al. [20], reflecting the anti-diabetic potential of linseed extract.

### 3.5. Anti-Inflammatory Activity of Linseed Extract

**Figure 8:**
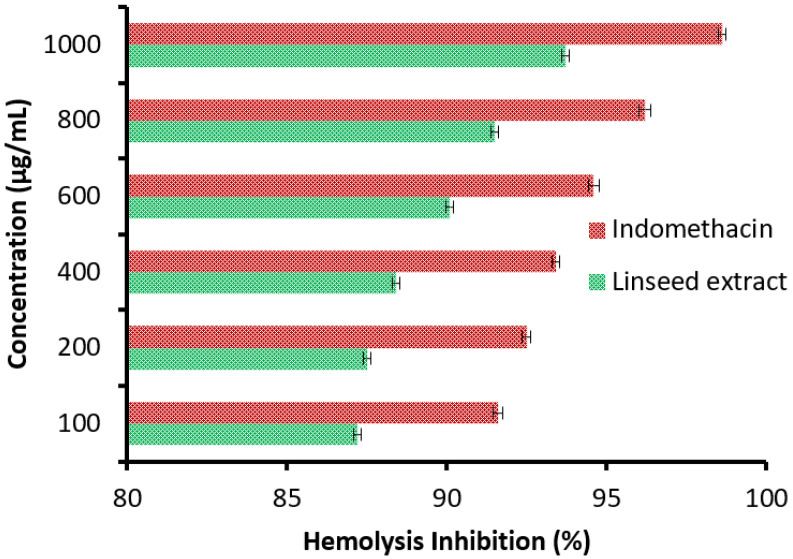
Anti-inflammatory activity of linseed extract via hemolysis inhibition measurement.

It was observed that hemolysis inhibition (%) increments with increasing linseed extract concentration in the range of 100 up to 1000 µg/mL (Figure 8), parallel with the positive control drug indomethacin. Hemolysis inhibition % was 87.2, 87.5, 88.4, 90.1, 91.5, and 93.7% utilizing linseed extract, while it was 91.6, 92.5, 93.4, 94.6, 96.2, and 98.6% utilizing indomethacin at 100, 200, 400, 600, 800, and 1000 µg/mL, respectively. The hemolysis inhibition tool was applied to assess the anti-inflammatory potential of linseed extract. In vivo, the anti-inflammatory and analgesic potentials of linseeds were confirmed [22]. The pharmacological uses of linseed were applied in vivo, where the topical application authenticated its healing properties, mainly in the diabetic animal model, via minimizing the inflammation stage, stimulating re-epithelialization development, improving neovascularization, and elevating the rate of surface closure [46]. In another report, Morsi et al. [47] investigated the activity of the phenolic and flavonoid contents of different linseed extracts for the management of inflammation, cancer, and oxidative stress.

### 3.6. Molecular Docking

**Figure 9:**
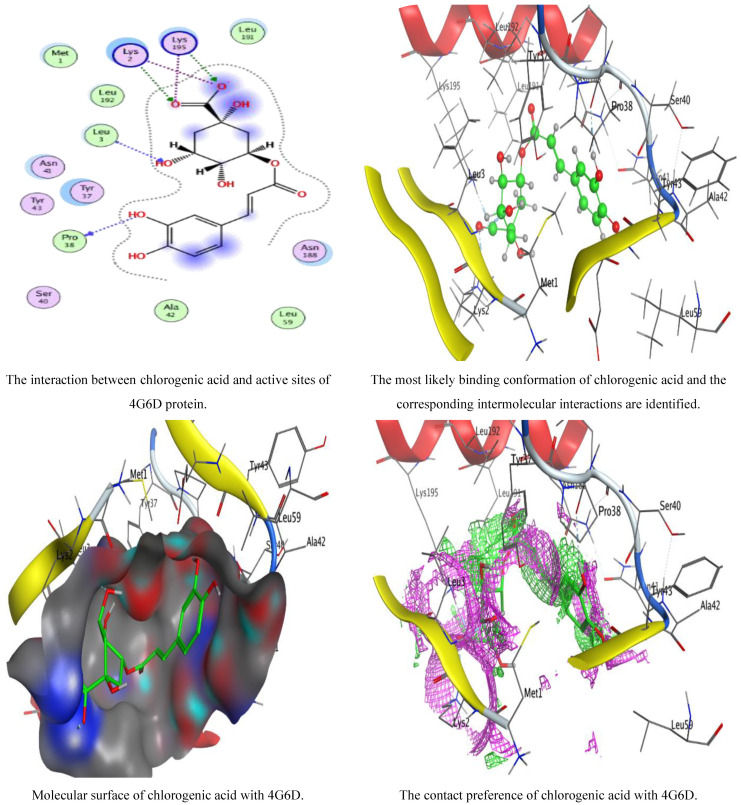

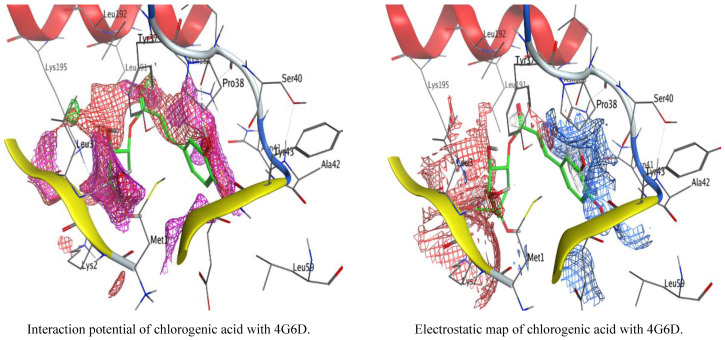
Molecular docking process of chlorogenic acid with 4G6D protein of *S. aureus*.

The widely used, computer-aided method for structural analysis and drug design is referred to as molecular docking. The goal of docking is to describe how two molecules, a ligand and a receptor, will bind to create a stable complex. The target binding site’s coordinate space is sampled during docking, and each potential ligand pose within that site is scored. The ligand posture with the highest score is then used to determine the binding mode for that substance. In our study, the ligand was permitted to be flexible to determine the proper conformations and produce structures with the smallest possible amount of energy. After docking, the optimal conformations of the ligand were examined for its binding interactions. The optimized structure of chlorogenic acid was docked into the selective pocket of *S. aureus* 4G6D. The score with the lowest energy, which indicates the best interactions between the ligand and the protein’s active site, was then recorded in Table 4. It was observed that chlorogenic acid demonstrated a favorable docking score (−6.26841 Kcal/mol) and interactions with the specified residues (PRO 38, LEU 3, LYS 195, and LYS 2) (Table 5, Figure 9). The root-mean-square deviation (RMSD) value was determined to be 1.0561 Å, demonstrating that our docking method is appropriate for the investigated inhibitor and that the MOE-Dock method is trusted for docking of this inhibitor. The observed negative score with the highest value of the free binding energy in the present investigation validated the antibacterial activity of chlorogenic acid against *S. aureus*, as mentioned in scientific studies that were similar but used different natural constituents [25,37,43,48]. Molecular docking interaction was used in several studies to confirm the action of biological activities against microbial pathogens such as *Proteus vulgaris* [28], *E.coli* [27], *S. aureus*, *C. albicans* [49], and *Salmonella typhi* [7].

## 4. Conclusions

This scientific paper indicates that several phenolic and flavonoid compounds were recorded in linseed extract via HPLC analysis. From the literature on the subject, it can be concluded that these compounds may differ according to the climatic and nutritional conditions of the plant cultivation. Inhibitory action, antibiofilm, and bacteriostatic activities against MRSA were documented using linseed extract. Most of the standard phenolic and flavonoid compounds, such as those detected in the linseed extract, inhibited the growth of MRSA, with different inhibition zones. The recorded MIC of the linseed extract was less than the MIC of the positive control, ciprofloxacin, indicating its efficacy against MRSA. Therefore, it is reasonable to conclude that bandages loaded with linseed extract could provide assistance in achieving inhibiting effects against topical MRSA. Evidence of biological activities, including anti-hemolysis and anti-diabetic activities, encourages the therapeutic application of linseed extract in managing several disorders of human health. The obtained findings of antioxidant potential lead to the recommendation that linseed extract might contribute to safe protection by preventing lipid peroxidation caused by free radicals. Further investigations are required in vivo to describe the biological activities and mechanistic actions of linseed extract to authenticate its therapeutic applications.

## Figures and Tables

**Table 1:** Flavonoid and phenolic compounds detected in linseed extract.

Constituent Name	Retention Time	Area	Area (%)	Concentration (µg/mL)
Unknown	2.55	58.50	8.05	Undetected
Unknown	2.65	217.16	29.90	Undetected
Gallic acid	3.35	33.41	4.60	155.10
Chlorogenic acid	4.21	262.44	36.14	1932.20
Catechin	4.78	6.60	0.91	87.85
Methyl gallate	5.61	96.88	13.34	284.31
Caffeic acid	6.06	7.93	1.09	32.78
Syringic acid	6.61	0.00	0.00	0.00
Pyrocatechol	6.78	0.00	0.00	0.00
Rutin	8.11	5.26	0.72	32.81
Ellagic acid	8.79	12.13	1.67	120.86
Coumaric acid	9.30	10.03	1.38	17.02
Vanillin	9.79	6.99	0.96	16.45
Ferulic acid	10.30	0.00	0.00	0.00
Naringenin	10.54	0.00	0.00	0.00
Daidzein	12.35	0.00	0.00	0.00
Querectin	12.85	0.00	0.00	0.00
Cinnamic acid	14.17	8.93	1.23	8.84
Apigenin	14.66	0.00	0.00	0.00
Kaempferol	15.17	0.00	0.00	0.00
Hesperetin	15.75	0.00	0.00	0.00

**Table 2:** Antibacterial activity of phenolic and flavonoid compounds against Methicillin-resistant *S. aureus*.

Compound	Inhibition Zone (mm)
Gallic acid	15.17 ± 0.29
Chlorogenic acid	17.67 ± 0.58
Catechin	12.84 ± 1.04
Methyl gallate	15.33 ± 1.15
Caffeic acid	14.83 ± 1.04
Rutin	15.17 ± 0.76
Ellagic acid	17.50 ± 0.87
Coumaric acid	10.67 ± 1.15
Vanillin	0.0 ± 0.00

**Table 3:** Antioxidant activity of linseed extract and ascorbic acid via DPPH method.

Concentration µg/mL	DPPH Scavenging %	
	Linseed Extract	Ascorbic Acid
Control	0.0 ± 0.00	0.0 ± 0.00
1.95	27.5 ± 0.004	40.2 ± 0.007
3.90	35.0 ± 0.002	44.3 ± 0.002
7.81	39.1 ± 0.004	54.7 ± 0.003
15.63	45.7 ± 0.005	62.6 ± 0.005
31.25	52.8 ± 0.003	69.6 ± 0.004
62.50	60.3 ± 0.004	76.4 ± 0.006
125	68.1 ± 0.002	84.9 ± 0.006
250	74.6 ± 0.006	91.1 ± 0.005
500	82.1 ± 0.001	92.9 ± 0.004
1000	89.9 ± 0.006	95.5 ± 0.002
IC_50_	20.8 µg/mL	4.81 µg/mL

**Table 4:** Docking scores and energies of chlorogenic acid with crystal structure of 4G6D protein of *S. aureus*.

mol	rseq	mseq	S	rmsd_refine	E_conf	E_place	E_score1	E_refine	E_score2
Chlorogenic acid	1	1	−6.26841	1.0561086	0.14604893	−50.8947	−14.5724	−31.1293	−6.26841
Chlorogenic acid	1	1	−5.89129	1.9759387	−2.8599455	−69.8904	−14.2878	−30.7783	−5.89129
Chlorogenic acid	1	1	−5.83516	2.193141	−0.15038154	−66.5634	−15.248	−29.6946	−5.83516
Chlorogenic acid	1	1	−5.71879	2.1018384	−1.6470002	−75.9115	−14.7519	−29.2169	−5.71879
Chlorogenic acid	1	1	−5.71228	1.7728372	−5.547338	−74.0203	−16.8466	−28.7861	−5.71228

**Table 5:** Interaction of chlorogenic acid with crystal structure of 4G6D protein of *S. aureus*.

Mol	Ligand	Receptor	Interaction	Distance	E (kcal/mol)
Chlorogenic acid	O 39	O PRO 38 (B)	H-donor	3.16	−1.6
	O 19	N LEU 3 (B)	H-acceptor	3.11	−1.6
	O 22	NZ LYS 195 (B)	H-acceptor	2.87	−2.8
	O 23	NZ LYS 2 (B)	H-acceptor	2.87	−2.3

## Data Availability

Not applicable.
